# Patient Portal Registrations at a Swiss Tertiary Referral Hospital Over the Course of the COVID-19 Pandemic: Retrospective Data Analysis

**DOI:** 10.2196/56961

**Published:** 2025-07-28

**Authors:** Anita D Linke, Amanda Franklin-Ryan, Angela Horn, Patrick E Beeler, Balthasar L Hug

**Affiliations:** 1 Faculty of Health Sciences and Medicine, University of Lucerne, Lucerne, Switzerland; 2 Center for Primary and Community Care, Faculty of Health Sciences and Medicine, University of Lucerne, Lucerne, Switzerland; 3 Department of General Internal Medicine, Cantonal Hospital of Lucerne, Spitalstrasse, Lucerne, 6000, Switzerland, 0041 412055102

**Keywords:** electronic patient portal, personal health record, MyChart, Epic system, COVID-19 pandemic, registration rate, registration pattern, age, gender

## Abstract

**Background:**

To enhance patient empowerment, the Cantonal Hospital of Lucerne launched a patient portal (MyChart) in December 2019, granting patients access to their medical records, diagnoses, and laboratory results. Months later, the first COVID-19 case was reported in Switzerland, with the pandemic dramatically affecting health care services.

**Objective:**

This analysis aims to investigate how the pattern of patient portal registrations evolved during the pandemic, with reference to the spread of COVID-19, as well as local and federal policies.

**Methods:**

This retrospective observational study analyzed the distribution of patient portal registrations after its introduction at the study site from December 1, 2019, until July 31, 2022. The descriptive analysis included the 7-day mean of registrations, plotted alongside the number of administered COVID-19 tests and COVID-19 vaccinations. This was analyzed concerning predefined time periods and stratified by age and gender. Additionally, an interrupted time series analysis was conducted for the different time periods.

**Results:**

A total of 126,519 patients registered on the patient portal during the study period, with a slightly higher proportion of female patients (n=66,118, 52.3%) and 11.3% (n=14,259) being 65 years of age or older. The daily registration rate differed substantially over the course of the COVID-19 pandemic, whereby four peaks with >200 registrations per day were identified. The first and third peaks coincide with high COVID-19 testing rates in autumn 2020 and 2021, whereas the second and fourth peaks coincide with the release of the vaccine in spring 2021 and the booster at the end of 2021. These patterns are also reflected in the interrupted time-series analysis: for every transition from one period to the next, the immediate effect of the intervention (level change) is statistically significant with *P*<.05. Regarding patient portal users aged 65 years or older, only two major peaks in registrations can be identified which coincide with the release of the COVID-19 vaccine and booster.

**Conclusions:**

The COVID-19 pandemic, with its disease dynamics, including testing and vaccinations, seems to have influenced the number of patient portal registrations. In addition, it appears that patients aged 65 years or older predominantly registered for COVID-19 vaccines.

## Introduction

Enhancing patient empowerment is one of the principal goals of Switzerland’s 2018‐2022 eHealth initiative 2.0 [1,2]. To improve transparency and promote shared decision-making, the Cantonal Hospital of Lucerne (called “study site” below) introduced a bespoke version of the MyChart smartphone and desktop app in December 2019 [3]. The patient portal MyChart allows patients almost full access to their medical records, including diagnoses and laboratory results [4,5].

Only a few months after the introduction of the patient portal, the first cases of COVID-19 were identified in Switzerland in February 2020 [6]. The dynamics of the disease itself, combined with precautions taken by the study site (eg, mandatory preconsultation questionnaires) and Swiss Federal Council policies including free COVID-19 testing [7], had a significant impact on Switzerland’s health care system [8].

Although MyChart has been widely adopted throughout the United States, there are smaller pilot projects in other countries, including the United Kingdom [9], the Netherlands [10], Norway [11], and Finland [12]. However, this is the first German version of the app [13], and this is a novel study investigating its registration pattern in Switzerland. To our knowledge, no research has been conducted on MyChart in German-speaking countries, and this study could prove useful for comparing patient portal use across cultures and health care models.

The purpose of this study was to quantify and characterize the number of patient portal registrations at the study site and compare them with the spread of COVID-19. Not only does it provide insights into the health-seeking behavior of different age groups and genders, but its fine-grained time-series analysis of electronic medical records tracks how this behavior evolved during the pandemic in response to local and federal policies. A detailed understanding of these behaviors could help better target patient portals to marginalized groups, increase patient engagement, and help coordinate health care provision in emergency situations.

## Methods

### Study Design

This retrospective observational study analyzed the distribution of patient portal registrations after its introduction at the study site. All registrations of patients between December 1, 2019, and July 31, 2022, were included.

### Setting and Participants

The Cantonal Hospital of Lucerne is a tertiary referral hospital based in Central Switzerland and is one of the largest hospital groups in the country. With facilities spread across three sites, it offers treatment to more than 48,500 inpatients and 920,000 outpatient contacts per year, with a catchment area of about 700,000 citizens [14].

MyChart was introduced after customization for the study site on December 1, 2019 [3]. After registering by signing a data release form, patients are granted broad access at no cost to their diagnoses, test results, and reports, as well as their prescribed medications. Further, patients are able to see their previous and upcoming appointments at the study site, update their allergies or medications, and complete any clinic-specific questionnaires [4,5]. Prior to MyChart, patients had to submit a request to the legal service team to view their medical records.

Patients were personally approached by their doctors to encourage MyChart registrations, and the platform was also promoted on the hospital website, with the implementation process managed by an internal team of physicians and IT professionals. To be eligible for patient portal registration at the study site, individuals must be patients aged 12 years or older. Alternatively, patients can choose to nominate a proxy to access health data on their behalf. For patients younger than 12 years of age, their caregivers can apply for proxy access to the minor’s account [4]. GPs are not automatically granted access to patient data and can only access it via another portal if additional written consent has been provided.

### Ethical Considerations

This study used completely anonymous data and conformed to the local law and the ethical review and research policies (Ethical Committee of Northwestern Switzerland EKNZ-ID REQ-2021‐00242). The authors adhered to the guidelines set forth by the RECORD (Reporting of studies Conducted using Observational Routinely-collected data) statement (Table S1 in Multimedia Appendix 1) [15].

### Data Collection

The anonymous data of the patient portal was extracted from the clinical data repository. The data contained information about patient portal user logins, their corresponding time stamps, and demographic data such as gender, age, and region of living. In addition, the first appointment at the study site after their registration and COVID-19 vaccination dates was extracted.

In order to investigate the course of the COVID-19 pandemic, additional data from the Swiss Federal Office of Public Health were used [16]. Extracted variables included the number of COVID-19 cases and administered antigen and polymerase chain reaction tests in the canton of Lucerne. A comparison of the number of administered vaccines reported on the patient portal and the total number of administered vaccines in the canton of Lucerne is visualized in Table S4 in Multimedia Appendix 1.

For the purposes of this study, the first login of a user was defined as a “registration.” Each login was defined as a “session,” whereas every action on the patient portal, like viewing diagnoses or test results, was described as an “action.”

### Data Analysis

Data preparation was performed by using SQL statements, while the software R (version 4.2.1; R Core Team) was used for data analysis and plotting. To analyze the distribution of daily registrations during the COVID-19 pandemic, the study period was divided into six time periods. In order to investigate possible correlations between the COVID-19 pandemic and the pattern of patient portal registrations, the 7-day mean of registrations was plotted alongside the number of administered COVID-19 vaccinations at the study site, as well as administered COVID-19 tests and the number of cases in the canton of Lucerne. A detailed chronological timeline (Table S2 in Multimedia Appendix 1) was used to annotate the visualizations with dates of the key events, including government measures and health events.

For further subgroup analyses, the 7-day mean of registrations was stratified by age and gender. Age was divided into two categories: patients being younger than 65 years of age or aged 65 years and older. The latter group is particularly important because adults older than 65 years of age were defined as high-risk groups for severe COVID-19 disease progression [17,18]. The analysis was again performed by comparison of graphs, but also differences in registration rate between the time periods.

To enhance the descriptive analysis, an interrupted time-series analysis focusing on the transitions between successive periods was conducted using the following formula:

7-day mean of registrations=β_0_+β_1_ * days_SincePortalRelease_+β_2_ * intervention+β_3_ * days_SinceIntervention_, whereas β_1_ reflects the slope of daily registrations before the intervention, β_2_ shows the level change (ie, immediate effect) of the intervention, and β_3_ represents the slope change (ie, sustained effect) after the intervention [19,20].

### Timeline of Main Events

**Figure 1.**
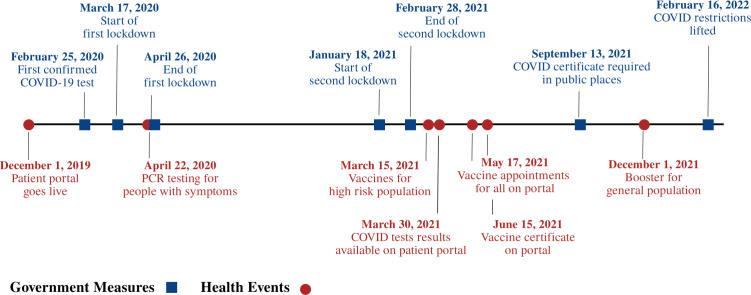
A chronological timeline outlines the main events of the COVID-19 pandemic in Switzerland and the introduction of the patient portal at the study site, categorized into government measures (highlighted in blue) and health-related events (highlighted in red). PCR: polymerase chain reaction.

Figure 1 shows a timeline containing the main events of the COVID-19 pandemic in Switzerland and the patient portal introduction at the study site. A detailed overview with all dates can be found in Table S2 in Multimedia Appendix 1.

## Results

### Time Periods

**Figure 2.**
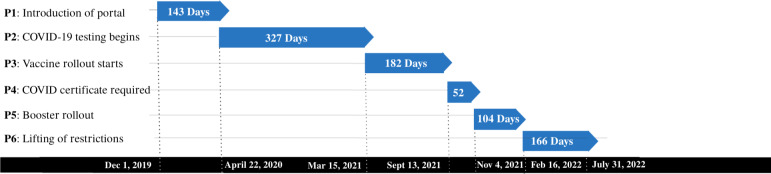
Predefined time periods, labeled P1 to P6, are established with specified start and end dates, along with their respective duration.

The study period from the introduction of the patient portal on December 1, 2019, until the data extraction on July 21, 2022, can be divided into six time periods (P1 to P6). As Figure 2 illustrates, the first period comprises a 4.5-month period from the introduction of the patient portal until the COVID-19 testing started in April 2020. The second stage covers an 11-month period from the beginning of the pandemic in April 2020 to the rollout of the vaccine for the high-risk population in March 2021, while the third period extends to September 2022 when a COVID-19 certificate (demonstrating vaccination or illness and recovery within a specified time period) was mandatory in public places. The fourth phase ends in November 2022 with the start of the booster for the high-risk population, while the next 4-month period extends to the lifting of the COVID-19 restrictions in February 2022. The final time period lasted until data extraction in July 2022.

### Participants

A total of 126,519 patients registered for the patient portal during the study period (Table 1). The users’ median age was 39.0 (IQR 26.0-55.0) years, with a slightly higher number of female users (n=66,118, 52.3%). Most patients lived in the canton of Lucerne (n=108,915, 86.1%), with the remainder distributed between Switzerland and Liechtenstein, or was unknown. Patients older than 65 years of age represented 11.3% (n=14,259) of total registrations, with these patients at increased risk of serious illness or mortality from SARS-CoV-2 infection [17,18].

**Table 1.** The characteristics of patient portal users are analyzed based on age groups.

	Overall (n=126,519)	Younger than 65 years of age (n=112,260)	Aged 65 years and older (n=14,259)	*P* value
Sex (male), n (%)	60,401 (47.7)	53,058 (47.3)	7343 (51.5)	<.001
Age (years), median (IQR)	39.00 (26.00-55.00)	36.00 (25.00-50.00)	71.00 (67.00-76.00)	<.001
Age categories (years), n (%)				<.001
Younger than 16 years	7828 (6.2)	7828 (7)	0 (0)	
16 to 30 years	34,841 (27.5)	34,841 (31)	0 (0)	
31 to 50 years	43,177 (34.1)	43,177 (38.5)	0 (0)	
51 to 64 years	26,414 (20.9)	26,414 (23.5)	0 (0)	
65 years and older	14,259 (11.3)	0 (0)	14,259 (100)	
Region of living (Canton Lucerne), n (%)	108,915 (86.1)	96,531 (86)	12,384 (86.9)	.005
Accessed by proxy access, n (%)	7841 (6.2)	7496 (6.7)	345 (2.4)	<.001
Time period of registration, n (%)				<.001
P1: Introduction of MyChart, 143 days	1066 (0.8)	952 (0.8)	114 (0.8)	
P2: COVID-19 testing begins, 327 days	36,959 (29.2)	33,924 (30.2)	3035 (21.3)	
P3: Vaccine rollout starts, 182 days	51,485 (40.7)	45,733 (40.7)	5752 (40.3)	
P4: COVID-19 certificate required, 52 days	13,876 (11)	12,502 (11.1)	1374 (9.6)	
P5: Booster rollout, 104 days	21,781 (17.2)	17,899 (15.9)	3882 (27.2)	
P6: Lifting of restrictions, 166 days	1352 (1.1)	1250 (1.1)	102 (0.7)	
MyChart sessions per year^a^, median (IQR)	10.90 (4.61-20.30)	11.04 (4.74-20.26)	9.71 (3.50-20.63)	<.001
Actions per MyChart session^b^, median (IQR)	21.13 (17.12-26.61)	21.00 (17.06-26.33)	22.47 (17.60-28.67)	<.001
First appointment type after registration, n (%)				<.001
No Appointments	38,054 (30.1)	35,041 (31.2)	3013 (21.1)	
1st or 2nd COVID-19 vaccination	41,225 (32.6)	37,990 (33.8)	3235 (22.7)	
3rd or 4th COVID-19 vaccination	9598 (7.6)	8087 (7.2)	1511 (10.6)	
Consultation or follow-up	10,720 (8.5)	8716 (7.8)	2004 (14.1)	
Others	26,922 (21.3)	22,426 (20)	4496 (31.5)	
Timespan until first appointment (days), median (IQR)	14.00 (1.00-96.00)	14.00 (1.00-105.00)	10.00 (1.00-53.00)	<.001
Appointments per year, n (%)				<.001
No appointments	38,054 (30.1)	35,041 (31.2)	3013 (21.1)	
1 to 4 appointments per year	65,943 (52.1)	59,353 (52.9)	6590 (46.2)	
5 or more appointments per year	22,522 (17.8)	17,866 (15.9)	4656 (32.7)	

a Number of logins of each user per year.

b Number of actions performed during one session (eg, view test results, view upcoming appointments, update their medications, or complete a clinic-specific questionnaire).

There was a total of 7846 (6.2%) proxy users, the majority of whom (n=6146, 78.5%) were aged 16 years or younger (Table S4 in Multimedia Appendix 1). The median number of patient portal sessions was 10.90 (IQR 4.61-20.30) per year, with median actions of 21.13 (IQR 17.12-26.61) per session. Just over half of the users (n=65,943, 52.1%) had 1 to 4 appointments at the study site per year. Further, 40.2% (n=50,823) of the first appointments after registration were related to receiving the COVID-19 vaccination or booster.

### All Registrations

**Figure 3.**
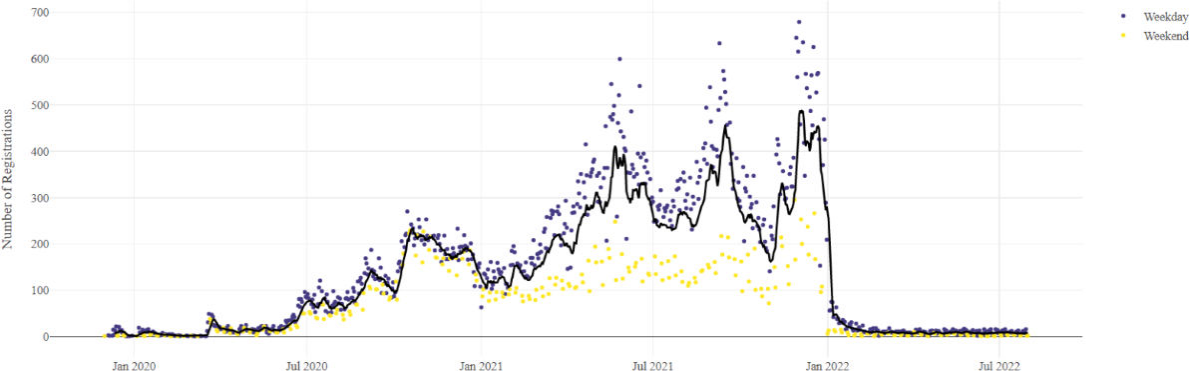
The 7-day mean of patient portal registrations is represented by a black line over the study period, while daily registrations on weekdays are shown as blue dots, and daily registrations on weekends are indicated by yellow dots.

Registrations rose steadily after the introduction of COVID-19 testing on April 22, 2020 (P2), and once again after the rollout of vaccines on March 15, 2021 (P3), before dropping suddenly prior to the relaxation of COVID-19 restrictions in February 2022 (Figure 3). Daily registration variability increased significantly after January 2021 and remained high throughout the vaccination period. The color coding in Figure 3 indicates that this variability is primarily attributable to a higher number of registrations on weekdays.

### Stratification by Time Period

**Figure 4.**
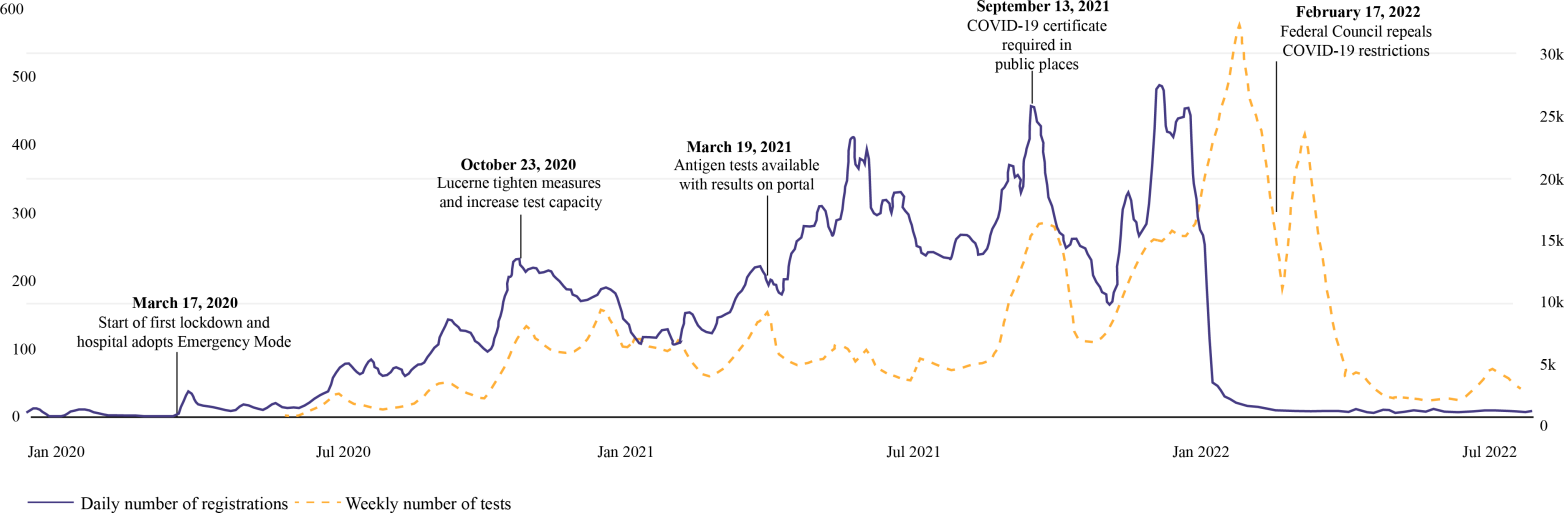
The 7-day mean of patient portal registrations (blue) and the 7-day mean of COVID-19 tests administered in the canton of Lucerne (orange), accompanied by main events regarding testing.

Figure S2 in Multimedia Appendix 1 illustrates this variation in more detail, showing the similarities in the distribution of registrations, vaccines, COVID-19 tests, and confirmed cases. In the first months after the patient portal introduction (P1-P2), the tests, registration, and case plots showed a similar trend, with a gradual increase following the introduction of COVID-19 testing. As Figure 4 shows, the registration rate appears to be tracking the test distribution from mid-June 2020 to the end of March 2021, with four peaks visible in both distributions during this period. Following the vaccine rollout in March 2021 (P3), the tests and registration distributions became decoupled, showing no further similarities except a single peak around September 13, 2021, and a general increase at the end of 2021 (P5).

Figure S1 in Multimedia Appendix 1 demonstrates substantial differences in daily registration patterns throughout the study period. Registrations started slowly prior to the pandemic (P1) and increased as COVID-19 tests were rolled out (P2), before rising sharply after the release of the first vaccine (P3 and P4). While registrations continued to rise in the first half of period 5 following the release of the booster, they subsequently fell rapidly in the remaining time period. This pattern is also reflected in the interrupted time-series analysis (Table 2 and Figure S7 in Multimedia Appendix 1). For every transition from one period to the next, the immediate effect of the intervention (level change) is statistically significant with *P*<.05. Further, there was a statistically significant sustained effect (slope change) for the transitions from P1 to P2 and P3 to P4, as well as from P5 to P6.

**Figure 5.**
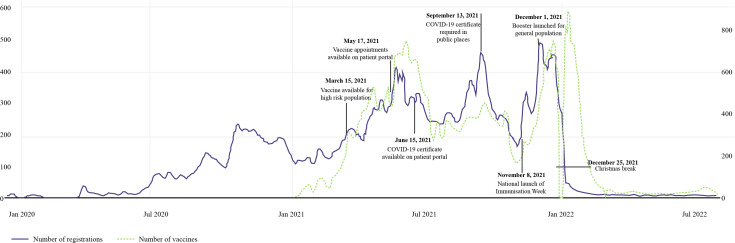
The 7-day mean of patient portal registrations (blue line) and the 7-day mean of COVID-19 vaccinations administered at the study site (green line), accompanied by main events regarding vaccines.

During periods 3 and 4, the registration distribution more closely resembled the vaccine distribution, showing clear peaks after the vaccine was released for the general population in mid-May 2021 and the booster was introduced on December 1, 2021 (Figure 5). Despite a rapid increase in the number of tests and cases throughout periods 5 and 6 (as of November 2022), daily registration rates dropped exponentially from 453 on December 22, 2021, to just 21 on January 22, 2022, and remained low (approximately 8 registrations a day) throughout period 6 (Table S4 in Multimedia Appendix 1).

**Table 2.** An interrupted time-series analysis is conducted for each transition between consecutive time periods.

		Coefficient	95% CI	*P* value
P1 versus P2				
Intercept		3.26	−10.90 to 17.41	.65
Slope (preintervention trend)		0.08	−0.08 to 0.24	.34
Level change (immediate effect)		15.71	0.91 to 30.50	.04
Slope change (sustained effect)		0.43	0.26 to 0.60	<.001
P2 versus P3				
Intercept		−41.43	−58.49 to −24.38	<.001
Slope (preintervention trend)		0.51	0.45 to 0.56	<.001
Level change (immediate effect)		46.87	30.11 to 63.62	<.001
Slope change (sustained effect)		−0.06	−0.20 to 0.08	.40
P3 versus P4	
Intercept		36.60	−40.97 to 114.17	.35
Slope (preintervention trend)		0.44	0.30 to 0.58	<.001
Level change (immediate effect)		60.00	29.23 to 90.77	<.001
Slope change (sustained effect)		−5.04	−5.95 to −4.14	<.001
P4 versus P5				
Intercept		3284.23	2107.27 to 4461.20	<.001
Slope (preintervention trend)		−4.46	−6.20 to −2.73	<.001
Level change (immediate effect)		299.07	237.15 to 360.99	<.001
Slope change (sustained effect)		−0.04	−1.88 to 1.79	.96
P5 versus P6				
Intercept		3724.30	[3391.25, 4057.35]	<.001
Slope (preintervention trend)		−4.65	[−5.09,‐4.21]	<.001
Level change (immediate effect)		36.25	[2.90, 69.60]	.03
Slope change (sustained effect)		4.64	[4.15, 5.14]	<.001

### Stratification by Age

Splitting the registration data into five age groups shows a similar trend for the 16‐ to 64-year-olds to the overall distribution (Figures3  6), with four clearly visible peaks. However, the first and third peaks, which appear to be associated with increased COVID-19 testing, become less pronounced with increasing age.

**Figure 6.**
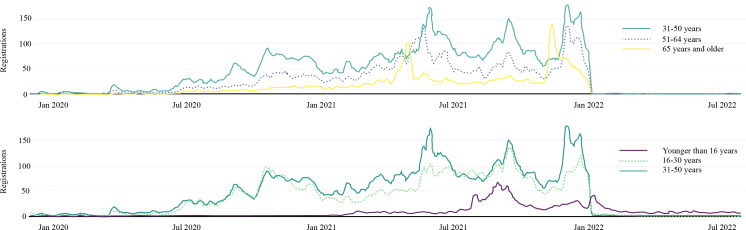
The 7-day mean of patient portal registrations grouped by age.

The younger than 16 years age group follows a completely different pattern, with a low registration rate until summer 2021, followed by a peak coinciding with the vaccine rollout for 12- to 15-year-olds starting on July 26, 2021. The second smaller peak might be related to the availability of vaccines for 5- to 12-year-olds after January 12, 2022.

The older than 65 years of age demographic showed no peak when the canton of Lucerne tightened restrictions in autumn 2020, and only two instead of three peaks during the COVID-19 vaccination period. This is also reflected in the interrupted time-series analysis, where the immediate effect (ie, level change) of the transition from P2 to P3 and P4 to P5 is statistically significant (*P*<.001) and quite large with 23.17 (95% CI 19.84-26.51), and 76.94 (95% CI 68.09-85.79) additional daily registrations, respectively (Table S6 and Figure S8 in Multimedia Appendix 1). Further, those older than 65 years of age had their highest mean daily registration rate after the booster rollout (P5).

### Stratification by Gender

**Figure 7.**
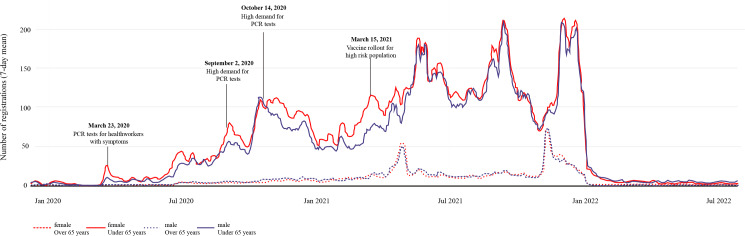
The 7-day mean of patient portal registrations grouped by gender and risk group. PCR: polymerase chain reaction.

Over the whole study period, more women than men (n=66,118, 52.3%) registered on the patient portal (Table 1). As Figure 7 illustrates, the distribution of registrations for men and women older than 65 years of age was very similar; however, in the younger than 65 years of age demographic, slightly more women registered between mid-October 2020 and April 2021. Following the release of vaccines on May 10, 2021, registration rates of both genders in the younger age group were almost equal and remained so until the end of the study period. Between January 2020 and spring 2021, there were several distinct peaks where female registrations among the younger than 65 years of age groups spiked suddenly, corresponding to the rollout and demand for COVID-19 tests and vaccines. Despite these minor differences, according to the interrupted time-series analysis, the registration rate of male and female patients developed similarly when transitions from one period to the next occurred (Table S7 and Figures S9 and S10 in Multimedia Appendix 1).

## Discussion

### Principal Findings

Between December 1, 2019, and July 31, 2022, over 126,000 patients registered on the patient portal. Initially, the registration pattern followed an expected course with a slow uptake, but subsequently appeared to be strongly influenced by the COVID-19 pandemic, with four peaks exceeding 200 registrations per day. The first and third peaks coincide with high COVID-19 testing rates, whereas the second and fourth peaks coincide with the release of the vaccine and booster. These patterns are also reflected in the interrupted time-series analysis: for every transition from one period to the next, the immediate effect of the intervention (level change) is statistically significant with *P*<.05. Among users aged 65 years or older, only two major peaks in registration can be identified, coinciding with the release of the COVID-19 vaccine and booster. Throughout the study period, slightly more women (n=66,118, 52.3%) registered on the patient portal.

### Comparison to Prior Work

#### Time Periods

In the 3 months following the launch of the patient portal on December 1, 2019, on average, there were fewer than 8 registrations a day (beginning of P1). Even with well-established patient portals, low registration rates are common, although 90% of US health care systems offer access to health data, only 15%‐30% of patients report accessing them [21].

However, on March 17, 2020, the Federal Council announced Switzerland’s first lockdown. Registrations rose rapidly after this announcement as the study site switched to emergency mode, reducing nonurgent care, replacing face-to-face appointments with web-based consultations, and providing remote monitoring of recovering patients with COVID-19 [22]. This increase continued throughout P2 after the introduction of COVID-19 testing and the availability of test results via the patient portal (Figure 4). A large fraction of registrations (n=13,535, 36.3%) were not accompanied by an appointment during this period or had a long time lag (median 134.5, IQR 22-300 days; Table S3 in Multimedia Appendix 1), suggesting many patients could have registered solely to view test results. Similar increases in both registrations and patient portal use were observed worldwide during the COVID-19 pandemic, with interest in telemedicine among both patients and doctors increasing dramatically. The number of children registered with one US University Hospital’s patient portal increased 10-fold immediately after the launch of a new enrollment strategy at the start of the pandemic [23], while researchers in Slovenia [24] and Saudi Arabia [25] reported similar trends. Canada’s University Health Network not only recorded an “overwhelming and rapid adoption” of its patient portal but also a registration pattern that “followed the emergence of each pandemic wave” [26].

Following the vaccine rollout, registrations seem to closely track the vaccine distribution during periods 3 and 5, with two clear peaks after the release of the first vaccine and booster (Figure 5). The distribution of appointments after registration mirrors this trend, with 53.9% (n=36,779) of first appointments related to a COVID-19 vaccine in P3 and 40.7% (n=8847) in P5. Vaccines continued at a steady rate at the end of P3 and throughout P4, rising in line with the number of tests and cases, peaking when the Federal Council demanded a valid COVID-19 certificate (requiring proof of recovery or vaccination) to enter public places. Interestingly, although tests increased significantly in the first half of P4, there was no associated rise in cases, perhaps indicating an increase in the number of healthy people taking tests to satisfy the certificate requirements, especially as this peak is absent in the older than 65 years of age demographic (Figure 6). The time lag between registration and first appointment was also reduced dramatically during the vaccine rollout periods to a median of 5 (IQR 0-28) days in P3 and 7 (IQR 0-29) days in P4 (Table S3 in Multimedia Appendix 1), implying patients may have registered to book a vaccine. The UK’s National Health Service app, granting patients the ability to make appointments, order prescriptions, and view medical records, followed a similar download pattern to this study site’s registration distribution, with peaks after the first lockdown and availability of proof of vaccination certificate on the portal [27].

In Period 5, both testing rates and cases rose dramatically as the highly infectious Omicron variant spread to Switzerland at the beginning of December 2021 (Table S2 in Multimedia Appendix 1). Concerned that the increase in cases could overwhelm intensive care units, the Federal Council imposed a series of further restrictions before relaxing measures on February 16, 2022 (Table S2 in Multimedia Appendix 1). Despite the increased impact of the COVID-19 pandemic on hospitals during this period, registration rates decreased exponentially from mid-December 2021 (Figure 4). Sudden decreases in registrations are unusual on patient portals, especially so soon after implementation. One Toronto hospital treating a similar number of patients to this study site recorded no attenuation in registrations even 8 years after its first launch [28]. Perhaps the sharp drop here could be a saturation effect: driven by the pandemic, patients may have been incentivized to register in order to book tests or vaccines and view the results; however, once all interested eligible patients had enrolled, new registrations decreased. Alternatively, portal promotion campaigns from individual hospital departments could also have influenced the distribution pattern. Although a large fraction of first appointments were linked to vaccines, more than 30% of patients booked a consultation, follow-up, or appointment with another department (Table S3 in Multimedia Appendix 1).

#### Age

As described in the result section, the age groups ranging from 16 to 64 years show four peaks, while those younger than 16 years and older than 65 years of age only had two peaks. Looking at the 16- to 64-year-olds, their first peak in autumn 2020 and their third peak in autumn 2021 seem to coincide with higher testing rates but are less pronounced with increasing age. These age differences are also visible in the testing rates across age groups in the canton of Lucerne (Figure S5 in Multimedia Appendix 1). The third peak coincides with a possible demand for COVID-19 certificates as they were required to access indoor areas of restaurants, as well as cultural and leisure facilities [29]. This presumably provided a stronger incentive for younger people to get tested.

Further, all adult age groups show a peak in registration rate during late spring or early summer 2021, coinciding with the rollout of vaccinations, but a time lag becomes visible. The older than 65 years of age group registrations peak first, followed by the 51- to 64-year-old age group, with peak registrations of the other two groups occurring almost simultaneously. This is consistent with prioritizing older than 75 years, 65 years, and then older than 55 years of age groups to get vaccinated first [30,31]. Later at the end of 2021 or the beginning of 2022, all age groups showed a peak in registration rate. Here, no gradual time lag is visible, and the older than 65 years of age group is followed directly by all other adult age groups, while the younger than 16 years of age group follows a little bit later. Here, the booster rollout for older than 65 years of age groups and the general population was only 2.5 weeks apart, providing a possible explanation for the missing time lag. The peak for the younger than 16 years of age group is probably due to the release of the first vaccinations for 5 to 12-year-olds.

The mean registration rate among the higher-risk older than 65 years age group demographic started low, with less than one registration per day during P1, respectively less than 10 registrations during P2. After the release of vaccines, the rate increased to a high level during P3 to P5 (31.6, 26.4, and 37.3 registrations per day), before dropping back to a mean of less than one registration per day during P6 (Table S4 in Multimedia Appendix 1). Therefore, the registration rate in the older than 65 years of age group actually did increase after the vaccines became available in March 2021 and increased even more after the booster rollout in November 2022.

Looking at the utilization of the patient portal, the older than 65 years of age group and the younger than 65 years of age group showed similar use intensity in regards to sessions per year, as well as actions per session (Table 1). This is in contrast to publications indicating that people younger than 65 years of age use patient portals more frequently than older patients [32,33]. This picture is reinforced by further studies showing that portal use increases with the number of chronic diagnoses, whereas this is mainly driven by younger patients [34-36]. These statements are only partly reflected in our data, where the use intensity is similar across all adult age groups (Table S4 in Multimedia Appendix 1), but the older than 65 years of age group has more appointments at the study site than users younger than 65 years of age (Table 1). Further, the use intensity increases remarkably with the number of appointments per year at the study site as a proxy for worse health status (Table S5 in Multimedia Appendix 1). This also stands in contrast to other studies stating that active users are healthier than inactive ones [37,38].

#### Gender

Researchers have consistently concluded that women are more likely to register for patient portals [39,40] and use telemedicine generally [41]. Although our data mirrors this trend in the younger than 65 years of age demographic during the first half of the study period (Figure 7), the overall gender difference is small, with 52.3% (n=66,118) female registrations compared to 60%‐70% in the literature [39-41]. Women typically spend more on health care and use more services [42]. However, during the pandemic, registrations appeared to have been influenced by testing and vaccination rates, and gender differences were reduced over the study period. However, this equalizing effect does not appear to be universal, with downloads of the UK National Health Service App still lowest in GP practices with the highest proportion of male patients, even at the height of the pandemic [27].

In the younger than 65 years of age group, registrations were slightly higher among women, perhaps corresponding to a higher female testing rate in the Swiss population (n=66,118, 52.3%) [43]; however, this difference almost disappeared after the vaccine rollout (Figure 7). Perhaps this is a saturation effect; women had already registered early and therefore did not need to register separately for vaccines. Alternatively, it could be an indication that Swiss women were more vaccine averse, with a 2020 Swiss survey reporting more than half of men were open to COVID-19 vaccination compared to 37% of women [44].

### Strengths and Limitations

To our knowledge, this is the only study site in the German-speaking context to have implemented MyChart [13], and this data analysis therefore adds valuable insights into the uptake of patient portals in Switzerland.

However, several limitations should be considered when interpreting the results of this study. First, it must be noted that the patient portal was launched only weeks before the start of the COVID-19 pandemic. This made it difficult to compare registration rates before and after, as there was no baseline pattern. Nonetheless, the study showed COVID-19 dynamics influencing the registration rate.

Second, this study is missing detailed patient information. Data were only available on patient portal users, making it impossible to compare them to nonusers and to identify factors predisposing patients to register. Due to this and the observational study design, a possible registration bias could not be addressed. Additionally, no data on diagnoses, treatments, and medical outcomes were available, and therefore, the analysis only referred to basic patient characteristics.

Third, it must be noted that there was only hospital-specific data available on COVID-19 vaccines, but not on COVID-19 testing. Consequently, data from the Federal Office of Public Health [16] was used for testing rates and case numbers in the canton of Lucerne.

### Future Directions

The focus of this study was to analyze the patient portal registration rate of a Swiss tertiary referral hospital. However, the interest lies not only in having a high number of users but in understanding by whom the portal is actively used and if this has the desired effect on patient engagement, communication with health care providers, and quality of care. Various researchers describe a relationship between higher portal utilization and patient characteristics like being younger than 65 years, female, employed, and a native speaker, as well as having a higher level of education and eHealth literacy [32-35,38]. With that in mind, it would be particularly interesting to investigate how the older than 65 years of age groups continued to use the patient portal in the long term. Research suggests that although this demographic often struggles to register on patient portals, once enrolled, they report finding them useful [45,46]. Further research could also perform subgroup analyses on use patterns and behavior based on age groups, number of appointments, or the involved departments at the study site.

Regarding the effect on health-related outcomes, the current literature draws mixed conclusions. It remains unclear whether being active on the patient portal during the time of a medical procedure is associated with a lower 30-day readmission rate and length of hospital stay, or if use actually might be associated with higher readmission rates, higher probability of an emergency department visit, and postoperative complications [32,37,47,48]. It could be instructive to study how patient portal utilization might be associated with health-related outcomes in the Swiss health care context.

It is further known that higher health literacy and ease and knowledge of how to use a portal, as well as provider encouragement, are facilitators of portal use [35,38,49]. Whereas lack of awareness of a patient portal, lack of training, limited technology access, privacy, and security concerns were identified as barriers to patient portal utilization [38,49-51]. This kind of information can only be obtained by applying qualitative and mixed methods research, as well as including users and nonusers in the study sample. This could lead to gaining a deeper understanding of the adoption of patient portals in the German-speaking context. It could also generate knowledge on how to address patients with low eHealth literacy in order to reduce their emerging disadvantages in health care engagement [52]. An additional focus could be laid on disparities in the registration and utilization of the patient portal across different socioeconomic groups, as a publication revealed health inequalities during the COVID-19 pandemic in Switzerland [43].

### Conclusions

The COVID-19 pandemic, with its disease dynamics, including testing and vaccinations, seems to have influenced the number of patient portal registrations. In addition, it appears that patients aged 65 years or older predominantly registered for COVID-19 vaccines.

## Supplementary material

10.2196/56961 Multimedia Appendix 1 Additional materials.

## Abbreviations

RECORD: Reporting of studies Conducted using Observational Routinely-collected data
