# Supplementary material for: Patient Portal Registrations at a Swiss Tertiary Referral Hospital Over the Course of the COVID-19 Pandemic: Retrospective Data Analysis
Source: J Med Internet Res. 2025 Jul 28;27:e56961. doi: 10.2196/56961 (PMC12303403; doi:10.2196/56961)
Supplement: Multimedia Appendix 1 [file jmir-v27-e56961-s001.pdf]

Patient portal registrations at a Swiss tertiary referral hospital over the course of the COVID-19 pandemic: A retrospective data analysis

**Authors:**	**ORCIDs:**
Anita D Linke[1*]	0009-0006-5747-3126
Amanda Franklin-Ryan[1*]	0009-0009-4549-7560
Angela Horn[1]	0009-0004-1200-0144
PD Dr. med. Patrick E Beeler[2]	0000-0002-6097-2480
Prof. Dr. med. Balthasar L Hug[2,3]	0000-0003-4235-1995

1 Faculty of Health Sciences and Medicine, University of Lucerne, Lucerne, Switzerland

2 Center for Primary and Community Care, Faculty of Health Sciences and Medicine, University of Lucerne, Lucerne, Switzerland

3 Department of General Internal Medicine, Cantonal Hospital Lucerne, Lucerne, Switzerland

* shared first authorship

Corresponding Author

Prof. Dr. med. Balthasar L Hug, Center for Primary and Community Care, Faculty of Health Sciences and Medicine, University of Lucerne, Lucerne, Switzerland, balthasar.hug@unilu.ch

Table of contents

Additional information on methodical aspects 3

RECORD statement 3

Detailed timeline of events 4

Additional descriptive analyses 7

Distribution of registrations, test, vaccines and cases 7

Distribution of registrations, vaccines and tests by age and gender 8

Distribution of COVID-19 vaccines and tests 9

Characteristics of patient portal users 10

Additional interrupted time series analyses 12

All patient portal registrations 12

Patient portal registrations grouped by age 13

Patient portal registrations grouped by gender 16

Additional information on methodical aspects

RECORD statement

Table S1. RECORD statement checklist for observational studies using routinely collected health data.

**Item**		**Referenced in chapter**
Title and Abstract		
1	1.1 Type of data	Abstract
	1.2 Geographic region and time frame	Abstract
	1.3 Linkage between databases	Not applicable
Introduction		
2	Background rationale	Introduction
3	Objectives	Introduction
Methods		
4	Study Design	Methods: Study design
5	Setting	Methods: Study design, Setting and subjects
6	Participants	
	6.1 Methods of study population selection	Methods: Setting and subjects, Data collection
	6.2 Validation of codes or algorithms	Not applicable
	6.3 Data linkage process	Not applicable
7	Variables	Not applicable
8	Data sources / management	Methods: Data collection
9	Bias	Discussion: Limitations and strengths
10	Study size	Methods: Data collection
11	Quantitative variables	Methods: Data analysis
12	Statistical methods	
	12.1 Data access	Methods: Data collection
	12.2 Cleaning methods	Not applicable
	12.3 Data linkage	Not applicable
Results		
13	Participants	Results: Participants
14	Descriptive data	Results: all subchapters
15	Outcome data	Results: All registrations, Stratification by time period, Stratification by age, Stratification by gender
16	Main results	Results: All registrations, Stratification by time period, Stratification by age, Stratification by gender
17	Other analyses	Results: Stratification by time period, Stratification by age, Stratification by gender<br>Appendix: Tables E-G, Figures E-G
Discussion		
18	Key results	Discussion: Main findings
19	Limitations	Discussion: Limitations and strengths
20	Interpretation	Discussion: Comparison with literature
21	Generalizability	Discussion: Limitations and strengths
Other information		
22	Funding	Statement on funding sources and conflicts of interest
-	Accessibility of protocol, raw data, and programming code	Code availability

Detailed timeline of events

Table S2. Chronological timeline of all mentioned events of the COVID-19 pandemic in Switzerland and the patient portal introduction at the study site.

Date	Event
01.12.2019	Patient portal MyChart going live [1]
25.02.2020	First confirmed Covid-19 case in Switzerland [2]
17.03.2020	Start of first lockdown [3]<br>Study site switches to emergency operations [4]
23.03.2020	PCR tests for all healthcare workers with symptoms [5]
03.04.2020	PCR tests for all symptomatic patients at study site [6]
22.04.2020	FOPH testing strategy: Generous testing of all patients and health care employees with at least one Covid-19 symptom [7,8]
26.04.2020	End of first lockdown [9]
02.09.2020	High demand for PCR tests (Figure 7)
14.10.2020	High demand for PCR tests (Figure 7)
23.10.2020	Canton of Lucerne increases restrictions and testing capacity [10]
23.12.2020	Vaccine available for high-risk population in the Canton of Lucerne [11]
06.01.2021	Start vaccination of employees with risk-factors or from exposed departments. Reuptake on February 22, 2021 following a supply shortage. No patients can be vaccinated [12–15]
18.01.2021	Start of second lockdown [16]
28.02.2021	End of second lockdown [16]
15.03.2021	Start vaccination of high-risk patients [17,18]<br>FOPH test offensive: The federal government covers all costs for antigen tests, only PCR tests for people without symptoms still have to be paid for by the patient [18]
29.03.2021	Antigen testing available at study site [19]
30.03.2021	Antigen and PCR test results available from patient portal [19]
03.05.2021	Start vaccination of over-55s in the canton of Lucerne [20]
10.05.2021	Start vaccination of general population in the canton of Lucerne [20]
17.05.2021	Start vaccination of general population at study site [N. Rösch, personal communication, March 11, 2024]<br>Vaccination appointments can be made via patient portal [21]
15.06.2021	Vaccination certificate on the patient portal [22]
26.07.2021	Vaccine available at study site for children between 12 and 16 years [23]<br>Start of walk-in vaccinations at study site [23]
13.09.2021	Covid certificate required in public places [24]
05.11.2021	Booster available for high risk population [25]
08.11.2021	Federal government and cantons launch national immunisation week [26]
01.12.2021	Booster launched for general population [27]
12.01.2022	Vaccine available at study site for children over 5 years [28]
17.02.2022	Relaxation of measures [29]

References

1. Cantonal Hospital of Lucerne. Meine Daten. n.d. Accessed January 5, 2024. https://www.luks.ch/ihr-luks/zukunftsprojekte/lukis-unser-spital-ist-digital/lukis-informationen-fuer-patienten/meine-daten

2. Federal Office of Public Health (FOPH). New Coronavirus 2019-nCoV: First confirmed case in Switzerland. February 25, 2020. Accessed February 4, 2024. https://www.bag.admin.ch/bag/en/home/das-bag/aktuell/medienmitteilungen.msg-id-78233.html

3. Federal Office of Public Health (FOPH). Coronavirus: Bundesrat erklärt die «ausserordentliche Lage» und verschärft die Massnahmen. March 17, 2020. Accessed February 4, 2024. https://www.bag.admin.ch/bag/de/home/das-bag/aktuell/medienmitteilungen.msg-id-78454.html

4. Cantonal Hospital of Lucerne. Coronavirus: LUKS wechselt vom Regel- in den Notfallbetrieb. March 16, 2020. Accessed February 4, 2024. https://www.luks.ch/newsroom/coronavirus-luks-wechselt-vom-regel-den-notfallbetrieb

5. Cantonal Hospital of Lucerne. Beschluss-Protokoll Nr. 10/20 der "Sitzung Sonderstab Pandemie" vom 23. März 2020.

6. Cantonal Hospital of Lucerne. Beschluss-Protokoll Nr. 21/20 der "Sitzung Sonderstab Pandemie" vom 3. April 2020.

7. Cantonal Hospital of Lucerne. Beschluss-Protokoll Nr. 38/20 der "Sitzung Sonderstab Pandemie" vom 22. April 2020.

8. Neue Strategie - BAG empfiehlt viel mehr Corona-Tests. *Schweizer Radio und Fernsehen (SRF)*. https://www.srf.ch/news/schweiz/neue-strategie-bag-empfiehlt-viel-mehr-corona-tests. April 22, 2020. Accessed March 4, 2024.

9. The Federal Council. Federal Council is gradually easing measures to protect against the new coronavirus. April 16, 2020. Accessed February 4, 2024. https://www.admin.ch/gov/en/start/documentation/media-releases.msg-id-78818.html

10. Kanton verschärft Massnahmen weiter. *Surseer Woche*. https://www.surseerwoche.ch/artikel/kanton-verschaerft-massnahmen-weiter. October 23, 2020. Accessed February 4, 2024.

11. Kanton Luzern startet mit Corona-Impfungen. *Surseer Woche*. http://www.surseerwoche.ch/artikel/kanton-luzern-startet-mit-corona-impfungen. December 21, 2020. Accessed February 4, 2024.

12. Cantonal Hospital of Lucerne. Beschluss-Protokoll Nr. 99 - Pandemie-Stab COVID-19 vom 8. Januar 2021.

13. Cantonal Hospital of Lucerne. Interne News-Meldung vom 15.01.2021. Published online January 15, 2021.

14. Cantonal Hospital of Lucerne. Interne News-Meldung vom 05.02.2021. Published online February 5, 2021.

15. Cantonal Hospital of Lucerne. Interne News-Meldung vom 22.02.2021. Published online February 22, 2021.

16. Parlamentsdienste. *Faktenbericht: Die Bundesversammlung und die Covid-19-Krise . Ein chronologischer Überblick. 24. Februar 2020 bis 18. Juni 2021.*; 2021:357. https://www.parlament.ch/centers/documents/_layouts/15/DocIdRedir.aspx?ID=DOCID-1-10033

17. Cantonal Hospital of Lucerne. Beschluss-Protokoll Nr. 112 - Pandemie-Stab COVID-19 vom 8. März 2021.

18. Cantonal Hospital of Lucerne. Beschluss-Protokoll Nr. 113 - Pandemie-Stab COVID-19 vom 15. März 2021.

19. Cantonal Hospital of Lucerne. Covid-19: Luzerner Kantonsspital führt auch Antigen-Schnelltests durch. March 30, 2021. Accessed February 4, 2024. https://www.luks.ch/newsroom/covid-19-luzerner-kantonsspital-fuehrt-auch-antigen-schnelltests-durch

20. Ab Mitte Mai starten im Kanton Luzern die Corona-Impfungen für alle – unabhängig der Altersgruppe. *Solothurner Zeitung*. https://www.solothurnerzeitung.ch/zentralschweiz/luzern/kanton-luzern-moderna-lieferung-bestaetigt-impfungen-der-uebrigen-bevoelkerung-beginnen-naechste-woche-ld.2131470. April 30, 2021. Accessed January 10, 2024.

21. Cantonal Hospital of Lucerne. Beschluss-Protokoll Nr. 123 - Pandemie-Stab COVID-19 vom 10. Mai 2021.

22. Cantonal Hospital of Lucerne. MeinLUKS ist bereit für das Covid-Impf-Zertifikat. June 15, 2021. Accessed February 4, 2024. https://www.luks.ch/newsroom/meinluks-ist-bereit-fuer-das-covid-impf-zertifikat

23. Luzerner Kantonsspital bietet neu Impftermine ohne Anmeldung an. *Luzerner Zeitung*. https://www.luzernerzeitung.ch/zentralschweiz/luzern/kanton-luzern-luzerner-kantonsspital-bietet-neu-impftermine-ohne-anmeldung-an-ld.2166440. July 23, 2021. Accessed February 4, 2024.

24. Federal Office of Public Health (FOPH). Coronavirus: Federal Council extends COVID certificate requirement and launches consultation on new entry rules. September 8, 2021. Accessed February 4, 2024. https://www.bag.admin.ch/bag/en/home/das-bag/aktuell/medienmitteilungen.msg-id-85035.html

25. Messmer M. Auffrisch-Impfung ist in Luzern ab sofort möglich. *Luzerner Zeitung*. https://www.luzernerzeitung.ch/zentralschweiz/luzern/schutz-vor-corona-auffrisch-impfung-ist-im-kanton-luzern-ab-sofort-moeglich-ld.2210488. November 5, 2021. Accessed February 4, 2024.

26. Federal Office of Public Health (FOPH). Coronavirus: Bund und Kantone lancieren die nationale Impfwoche. November 3, 2021. Accessed February 4, 2024. https://www.bag.admin.ch/bag/de/home/das-bag/aktuell/medienmitteilungen.msg-id-85720.html

27. Ab Dezember bekommen im Kanton Luzern alle Volljährigen den Booster auch spontan. *Luzerner Zeitung*. https://www.luzernerzeitung.ch/zentralschweiz/luzern/coronapandemie-auffrischimpfung-fuer-unter-65-jaehrige-ab-1-dezember-2021-verfuegbar-ld.2221517. November 29, 2021. Accessed February 4, 2024.

28. Cantonal Hospital of Lucerne. Erste Kinder am Luzerner Kantonsspital gegen Covid-19 geimpft. January 12, 2022. Accessed February 4, 2024. https://www.luks.ch/newsroom/erste-kinder-am-luzerner-kantonsspital-gegen-covid-19-geimpft

29. Federal Office of Public Health (FOPH). Coronavirus: Federal Council to lift measures – mask requirement on public transport and in healthcare institutions and isolation in the event of illness to remain until end of March. February 16, 2022. Accessed February 4, 2024. https://www.bag.admin.ch/bag/en/home/das-bag/aktuell/medienmitteilungen.msg-id-87216.html

Additional descriptive analyses

Distribution of registrations, test, vaccines and cases

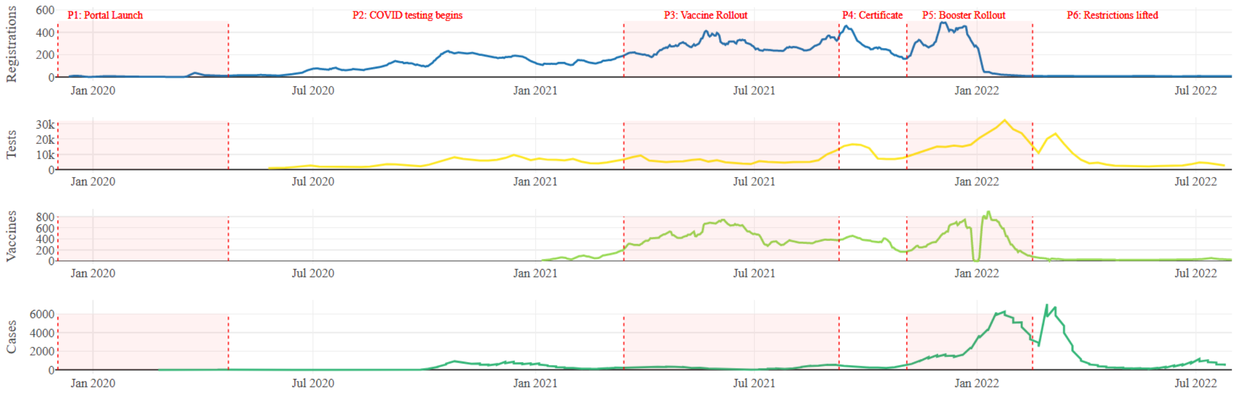

Figure S1. i) Daily Number of patient portal registrations (7-day mean), ii) Weekly Number of administered COVID-19 tests in the canton of Lucerne, iii) Daily Number of administered COVID-19 vaccinations at study site (7-day mean), iv) Weekly Number of confirmed COVID-19 cases in the canton of Lucerne.

Distribution of registrations, vaccines and tests by age and gender

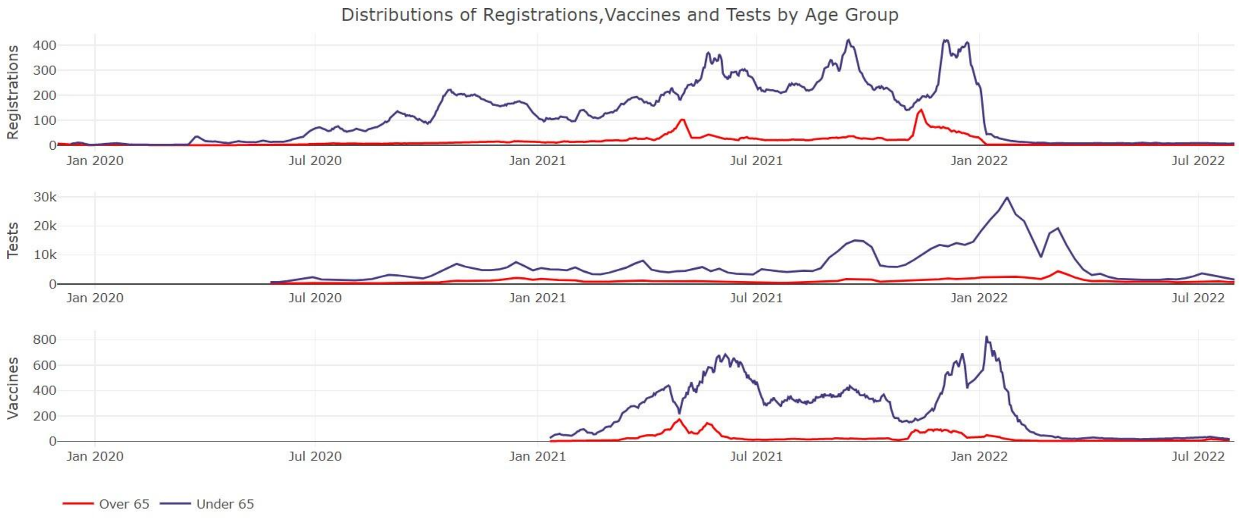

Figure S2. All figures are grouped by age group, i) 7-day-mean of patient portal registrations, ii) 7-day-mean of administered COVID-19 tests in the canton of Lucerne, iii) 7-day-mean of administered COVID-19 vaccinations reported on patient portal.

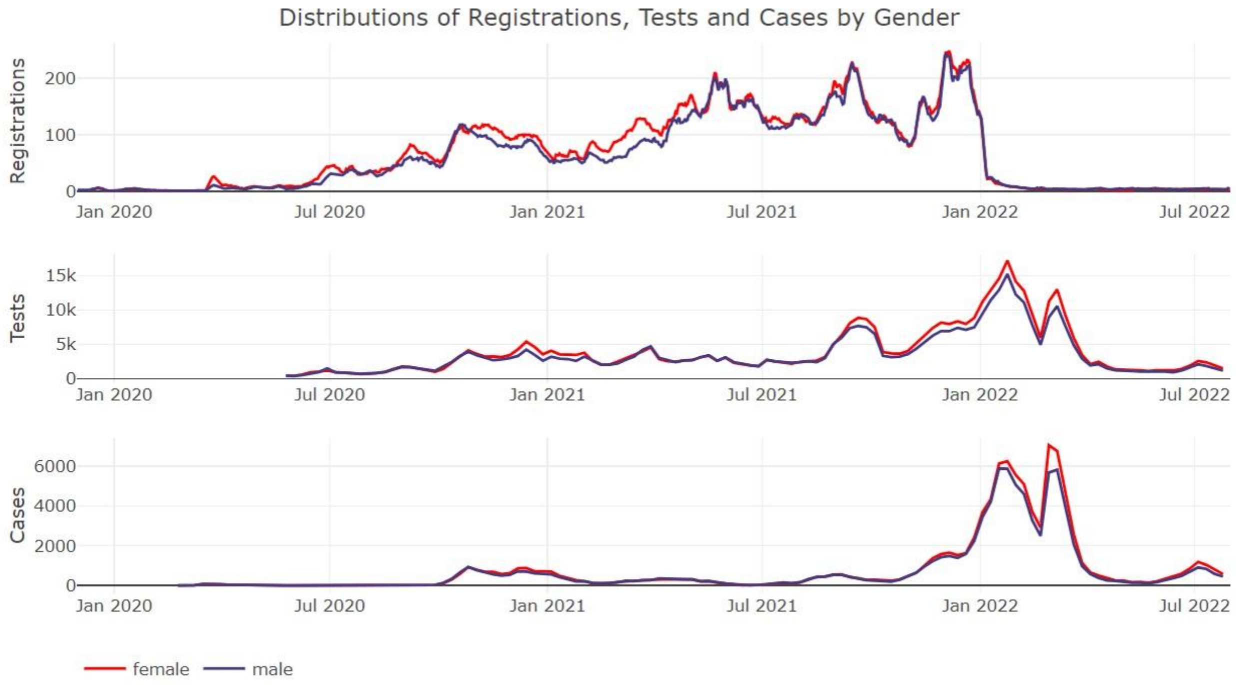

Figure S3. All figures are grouped by gender, i) 7-day-mean of patient portal registrations, ii) 7-day-mean of administered COVID-19 tests in the canton of Lucerne, iii) 7-day-mean of COVID-19 cases (weekly BAG data).

Distribution of COVID-19 vaccines and tests

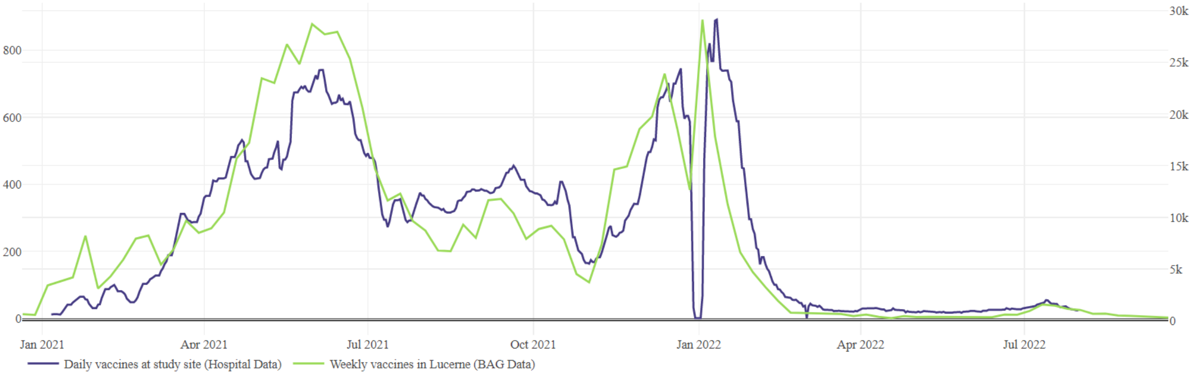

Figure S4. Distribution of COVID-19 vaccines at the study site (patient portal data 7-day mean) and in canton of Lucerne (weekly BAG data).

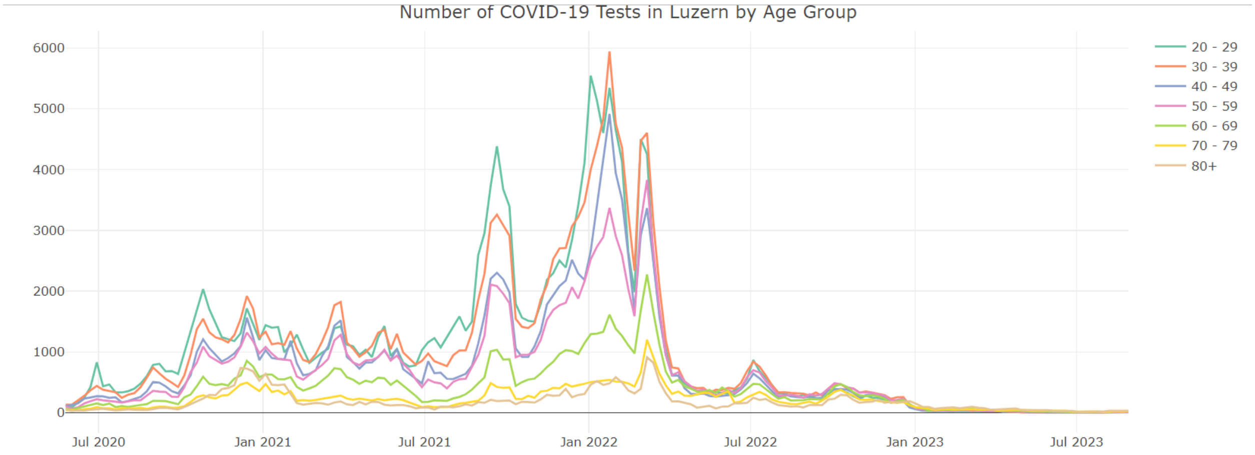

Figure S5. Number of COVID-19 tests administered in the canton of Lucerne stratified by age.

Characteristics of patient portal users

Table S3. Characteristics of patient portal users stratified by time period of registration (P1 to P6). The values in P6 regarding portal utilisation and first appointment after registration are displayed for transparency reasons, but should be treated with caution due to the short observation period.

	Overall	P1	P2	P3	P4	P5	P6	*P*
n	126519	1066	36959	51485	13876	21781	1352	
Gender, male (%)	60401 (47.7)	443 (41.6)	16801 (45.5)	24821 (48.2)	6875 (49.5)	10716 (49.2)	745 (55.1)	<0.001
Age, median years [IQR]	39.00 [26.00, 55.00]	41.00 [31.00, 55.00]	37.00 [27.00, 52.00]	40.00 [26.00, 56.00]	36.00 [24.00, 53.00]	43.00 [28.00, 59.00]	9.00 [3.00, 15.00]	<0.001
Age categories (%)								<0.001
Younger than 16 years	7828 (6.2)	1 (0.1)	349 (0.9)	3185 (6.2)	1203 (8.7)	2036 (9.3)	1054 (78.0)	
16 to 30 years	34841 (27.5)	249 (23.4)	12137 (32.8)	13723 (26.7)	4096 (29.5)	4570 (21.0)	66 (4.9)	
31 to 50 years	43177 (34.1)	464 (43.5)	14121 (38.2)	17118 (33.2)	4710 (33.9)	6691 (30.7)	73 (5.4)	
51 to 64 years	26414 (20.9)	238 (22.3)	7317 (19.8)	11707 (22.7)	2493 (18.0)	4602 (21.1)	57 (4.2)	
65 years and older	14259 (11.3)	114 (10.7)	3035 (8.2)	5752 (11.2)	1374 (9.9)	3882 (17.8)	102 (7.5)	
MyChart sessions per year, median [IQR]	10.90 [4.61, 20.30]	11.72 [4.34, 23.00]	8.45 [3.46, 17.72]	12.69 [5.87, 21.15]	12.24 [5.71, 22.78]	9.07 [4.39, 17.92]	74.09 [29.13, 194.49]	<0.001
Actions per MyChart session, median [IQR]	21.13 [17.12, 26.61]	20.38 [16.96, 24.15]	19.55 [15.88, 24.05]	21.25 [17.69, 26.10]	21.14 [16.79, 26.80]	24.00 [18.64, 32.00]	36.77 [27.24, 52.27]	<0.001
First appointment type after registration (%)								<0.001
No Appointments	38054 (30.1)	207 (19.4)	13535 (36.6)	11252 (21.9)	4326 (31.2)	7714 (35.4)	1020 (75.4)	
1st or 2nd Covid-19 vaccination	41225 (32.6)	184 (17.3)	7152 (19.4)	25527 (49.6)	5311 (38.3)	3041 (14.0)	10 (0.7)	
3rd or 4th Covid-19 vaccination	9598 (7.6)	6 (0.6)	1046 (2.8)	2197 (4.3)	514 (3.7)	5806 (26.7)	29 (2.1)	
Consultation or Follow-up	10720 (8.5)	199 (18.7)	4243 (11.5)	3733 (7.3)	1035 (7.5)	1446 (6.6)	64 (4.7)	
Others	26922 (21.3)	470 (44.1)	10983 (29.7)	8776 (17.0)	2690 (19.4)	3774 (17.3)	229 (16.9)	
Timespan until first appointment, median days [IQR]	14.00 [1.00, 96.00]	134.00 [26.50, 373.00]	134.50 [22.00, 300.00]	5.00 [0.00, 28.00]	7.00 [0.00, 29.00]	13.00 [2.00, 29.00]	11.00 [1.00, 38.25]	<0.001
Appointments per year (%)								<0.001
No appointments	38054 (30.1)	207 (19.4)	13535 (36.6)	11252 (21.9)	4326 (31.2)	7714 (35.4)	1020 (75.4)	
1 to 4 appointments per year	65943 (52.1)	527 (49.4)	16752 (45.3)	31025 (60.3)	7134 (51.4)	10407 (47.8)	98 (7.2)	
5 and more appointments per year	22522 (17.8)	332 (31.1)	6672 (18.1)	9208 (17.9)	2416 (17.4)	3660 (16.8)	234 (17.3)	

Table S4. Characteristics of patient portal users stratified by age groups.

	Overall	Younger than 16 years	16 to 30 years	31 to 50 years	51 to 64 years	65 years and older	*P*
n	126519	7828	34841	43177	26414	14259	
Average registrations per day	129.9	8.0	35.8	44.3	27.1	14.6	not tested
Gender, male (%)	60401 (47.7)	4222 (53.9)	15609 (44.8)	20646 (47.8)	12581 (47.6)	7343 (51.5)	<0.001
Accessed by proxy access (%)	7841 (6.2)	6146 (78.5)	518 (1.5)	525 (1.2)	307 (1.2)	345 (2.4)	<0.001
Time period of registration, n (%)							<0.001
P1: Introduction of MyChart, 143 days	1066 (0.8)	1 (0.0)	249 (0.7)	464 (1.1)	238 (0.9)	114 (0.8)	
P2: COVID-19 testing begins, 327 days	36959 (29.2)	349 (4.5)	12137 (34.8)	14121 (32.7)	7317 (27.7)	3035 (21.3)	
P3: Vaccine rollout starts, 182 days	51485 (40.7)	3185 (40.7)	13723 (39.4)	17118 (39.6)	11707 (44.3)	5752 (40.3)	
P4: Covid certificate required , 52 days	13876 (11.0)	1203 (15.4)	4096 (11.8)	4710 (10.9)	2493 (9.4)	1374 (9.6)	
P5: Booster rollout, 104 days	21781 (17.2)	2036 (26.0)	4570 (13.1)	6691 (15.5)	4602 (17.4)	3882 (27.2)	
P6: Lifting of restrictions, 166 days	1352 (1.1)	1054 (13.5)	66 (0.2)	73 (0.2)	57 (0.2)	102 (0.7)	
Time period of registration, average registrations per day							not tested
P1: Introduction of MyChart, 143 days	7.5	0.0	1.7	3.2	1.7	0.8	
P2: COVID-19 testing begins, 327 days	113.0	1.1	37.1	43.2	22.4	9.3	
P3: Vaccine rollout starts, 182 days	282.9	17.5	75.4	94.1	64.3	31.6	
P4: Covid certificate required , 52 days	266.8	23.1	78.8	90.6	47.9	26.4	
P5: Booster rollout, 104 days	209.4	19.6	43.9	64.3	44.3	37.3	
P6: Lifting of restrictions, 166 days	8.1	6.3	0.4	0.4	0.3	0.6	
MyChart sessions per year, median [IQR]	10.90 [4.61, 20.30]	23.03 [5.20, 51.58]	10.73 [4.72, 18.82]	10.66 [4.73, 19.16]	10.66 [4.73, 19.88]	9.71 [3.50, 20.63]	<0.001
Actions per MyChart session, median [IQR]	21.13 [17.12, 26.61]	48.96 [25.13, 72.19]	19.33 [16.06, 23.24]	21.04 [17.45, 25.60]	22.11 [18.07, 27.33]	22.47 [17.60, 28.67]	<0.001
First appointment type after registration (%)							<0.001
No Appointments	38054 (30.1)	3656 (46.7)	11938 (34.3)	13198 (30.6)	6249 (23.7)	3013 (21.1)	
1st or 2nd Covid-19 vaccination	41225 (32.6)	1508 (19.3)	13051 (37.5)	14654 (33.9)	8777 (33.2)	3235 (22.7)	
3rd or 4th Covid-19 vaccination	9598 (7.6)	989 (12.6)	1817 (5.2)	3093 (7.2)	2188 (8.3)	1511 (10.6)	
Consultation or Follow-up	10720 (8.5)	502 (6.4)	2136 (6.1)	3247 (7.5)	2831 (10.7)	2004 (14.1)	
Others	26922 (21.3)	1173 (15.0)	5899 (16.9)	8985 (20.8)	6369 (24.1)	4496 (31.5)	
Timespan until first appointment, median days [IQR]	14.00 [1.00, 96.00]	25.00 [3.00, 108.00]	17.00 [1.00, 148.00]	14.00 [1.00, 102.00]	12.00 [1.00, 67.00]	10.00 [1.00, 53.00]	<0.001
Appointments per year (%)							<0.001
No appointments	38054 (30.1)	3656 (46.7)	11938 (34.3)	13198 (30.6)	6249 (23.7)	3013 (21.1)	
1 to 4 appointments per year	65943 (52.1)	3104 (39.7)	18824 (54.0)	23043 (53.4)	14382 (54.4)	6590 (46.2)	
5 and more appointments per year	22522 (17.8)	1068 (13.6)	4079 (11.7)	6936 (16.1)	5783 (21.9)	4656 (32.7)	

Table S5. Characteristics of patient portal users stratified by number of appointments per year at study site.

	**Overall**	**No appointments**	**1 to 4 appointments per year**	**5 and more appointments per year**	***P***
n	126519	38054	65943	22522	
Gender, male (%)	60401 (47.7)	20129 (52.9)	30769 (46.7)	9503 (42.2)	<0.001
Age, median years [IQR]	39.00 [26.00, 55.00]	35.00 [24.00, 50.00]	39.00 [27.00, 55.00]	48.00 [32.00, 62.00]	<0.001
Age categories (%)					<0.001
Younger than 16 years	7828 (6.2)	3656 (9.6)	3104 (4.7)	1068 (4.7)	
16 to 30 years	34841 (27.5)	11938 (31.4)	18824 (28.5)	4079 (18.1)	
31 to 50 years	43177 (34.1)	13198 (34.7)	23043 (34.9)	6936 (30.8)	
51 to 64 years	26414 (20.9)	6249 (16.4)	14382 (21.8)	5783 (25.7)	
65 years and older	14259 (11.3)	3013 (7.9)	6590 (10.0)	4656 (20.7)	
Accessed by proxy access (%)	7841 (6.2)	3894 (10.2)	2813 (4.3)	1134 (5.0)	<0.001
Time period of registration (%)					<0.001
P1: Introduction of MyChart, 143 days	1066 (0.8)	207 (0.5)	527 (0.8)	332 (1.5)	
P2: COVID-19 testing begins, 327 days	36959 (29.2)	13535 (35.6)	16752 (25.4)	6672 (29.6)	
P3: Vaccine rollout starts, 182 days	51485 (40.7)	11252 (29.6)	31025 (47.0)	9208 (40.9)	
P4: Covid certificate required , 52 days	13876 (11.0)	4326 (11.4)	7134 (10.8)	2416 (10.7)	
P5: Booster rollout, 104 days	21781 (17.2)	7714 (20.3)	10407 (15.8)	3660 (16.3)	
P6: Lifting of restrictions, 166 days	1352 (1.1)	1020 (2.7)	98 (0.1)	234 (1.0)	
MyChart sessions per year, median [IQR]	10.90 [4.61, 20.30]	4.81 [2.33, 10.17]	12.31 [6.55, 19.41]	23.26 [12.30, 40.37]	<0.001
Actions per MyChart session, median [IQR]	21.13 [17.12, 26.61]	19.38 [15.00, 27.00]	21.14 [17.73, 25.81]	23.17 [19.27, 28.49]	<0.001
First appointment type after registration (%)					<0.001
No Appointments	38054 (30.1)	38054 (100.0)	0 (0.0)	0 (0.0)	
1st or 2nd Covid-19 vaccination	41225 (32.6)	0 (0.0)	36217 (54.9)	5008 (22.2)	
3rd or 4th Covid-19 vaccination	9598 (7.6)	0 (0.0)	8834 (13.4)	764 (3.4)	
Consultation or Follow-up	10720 (8.5)	0 (0.0)	6061 (9.2)	4659 (20.7)	
Others	26922 (21.3)	0 (0.0)	14831 (22.5)	12091 (53.7)	
Timespan until first appointment, median days [IQR]	14.00 [1.00, 96.00]	NA	16.00 [1.00, 132.00]	10.00 [1.00, 43.00]	<0.001

Additional interrupted time series analyses

All patient portal registrations

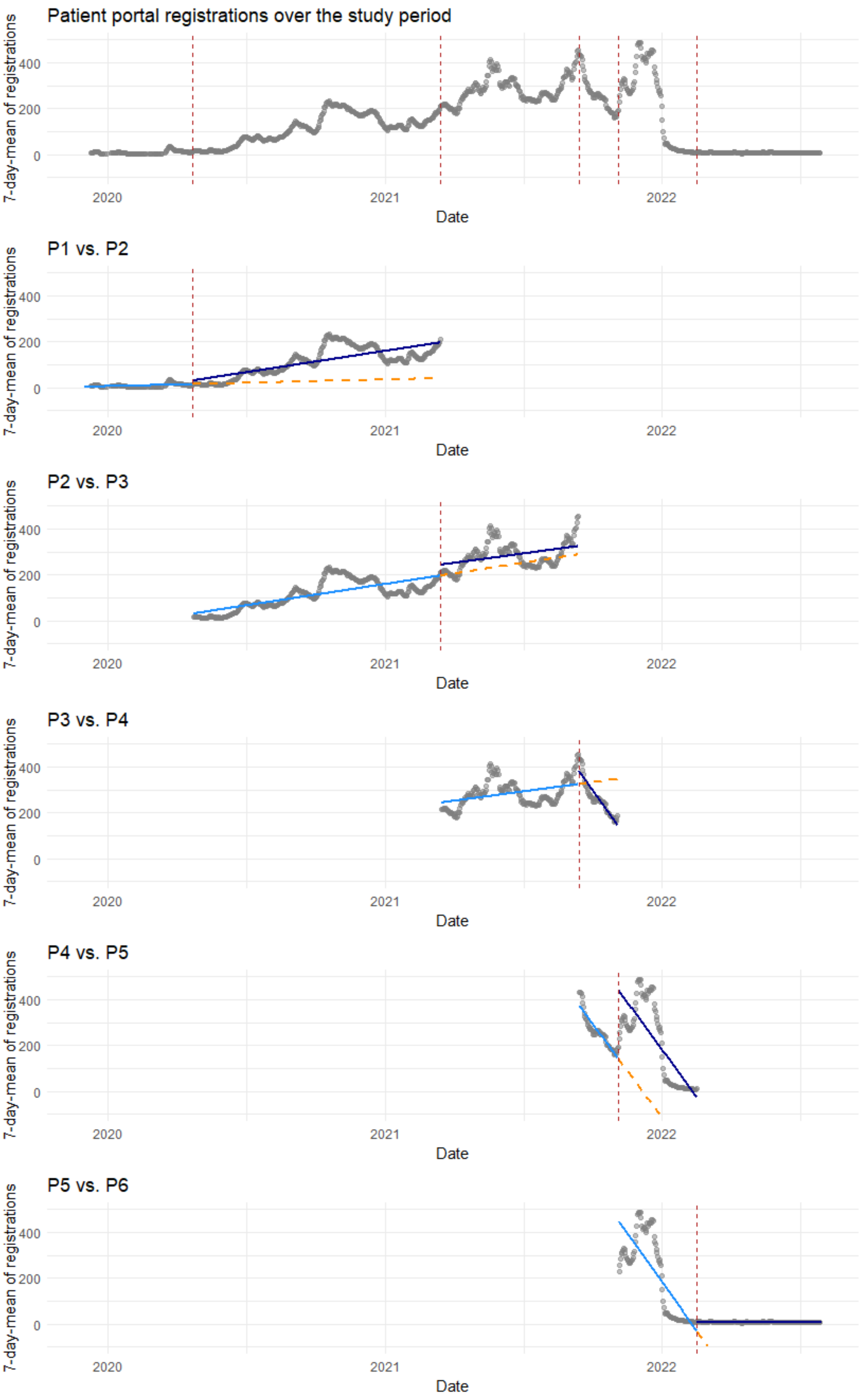

Figure S6. Interrupted time series analysis for each transition from one period to the next.

Patient portal registrations grouped by age

Table S6. Interrupted time series analysis for each transition from one period to the next grouped by age group.

		Under 65			65 and older		
		Coefficient	95% CI	*P*	Coefficient	95% CI	*P*
P1 vs. P2	Intercept	1.89	[-11.93, 15.72]	0.79	1.36	[0.73, 2.00]	< .001
	Slope (pre-intervention trend)	0.08	[-0.08, 0.24]	0.30	0.00	[-0.01, 0.00]	0.19
	Level change (immediate effect)	16.13	[1.69, 30.57]	0.03	-0.43	[-1.09, 0.24]	0.21
	Slope change (sustained effect)	0.37	[0.20, 0.53]	< .001	0.06	[0.05, 0.07]	< .001
P2 vs. P3	Intercept	-33.81	[-50.01, -17.61]	< .001	-7.63	[-11.02, -4.23]	< .001
	Slope (pre-intervention trend)	0.45	[0.40, 0.50]	< .001	0.06	[0.04, 0.07]	< .001
	Level change (immediate effect)	23.7	[7.78, 39.61]	0.004	23.17	[19.84, 26.51]	< .001
	Slope change (sustained effect)	0.10	[-0.03, 0.23]	0.12	-0.16	[-0.19, -0.13]	< .001
P3 vs. P4	Intercept	-56.6	[-128.11, 14.92]	0.12	93.20	[71.93, 114.47]	< .001
	Slope (pre-intervention trend)	0.55	[0.42, 0.68]	< .001	-0.11	[-0.15, -0.07]	< .001
	Level change (immediate effect)	51.22	[22.85, 79.59]	< .001	8.78	[0.34, 17.22]	0.04
	Slope change (sustained effect)	-4.98	[-5.81, -4.14]	< .001	-0.06	[-0.31, 0.18]	0.61
P4 vs. P5	Intercept	3147.44	[1988.44, 4306.44]	< .001	136.79	[-31.42, 305.00]	0.11
	Slope (pre-intervention trend)	-4.30	[-6.01, -2.59]	< .001	-0.16	[-0.41, 0.08]	0.19
	Level change (immediate effect)	222.13	[161.16, 283.11]	< .001	76.94	[68.09, 85.79]	< .001
	Slope change (sustained effect)	0.96	[-0.84, 2.77]	0.29	-1.01	[-1.27, -0.75]	< .001
P5 vs. P6	Intercept	2775.28	[2443.26, 3107.30]	< .001	949.02	[903.68, 994.35]	< .001
	Slope (pre-intervention trend)	-3.44	[-3.88, -3.00]	< .001	-1.21	[-1.27, -1.15]	< .001
	Level change (immediate effect)	11.42	[-21.83, 44.67]	0.50	24.83	[20.29, 29.37]	< .001
	Slope change (sustained effect)	3.44	[2.95, 3.93]	< .001	1.21	[1.14, 1.27]	< .001

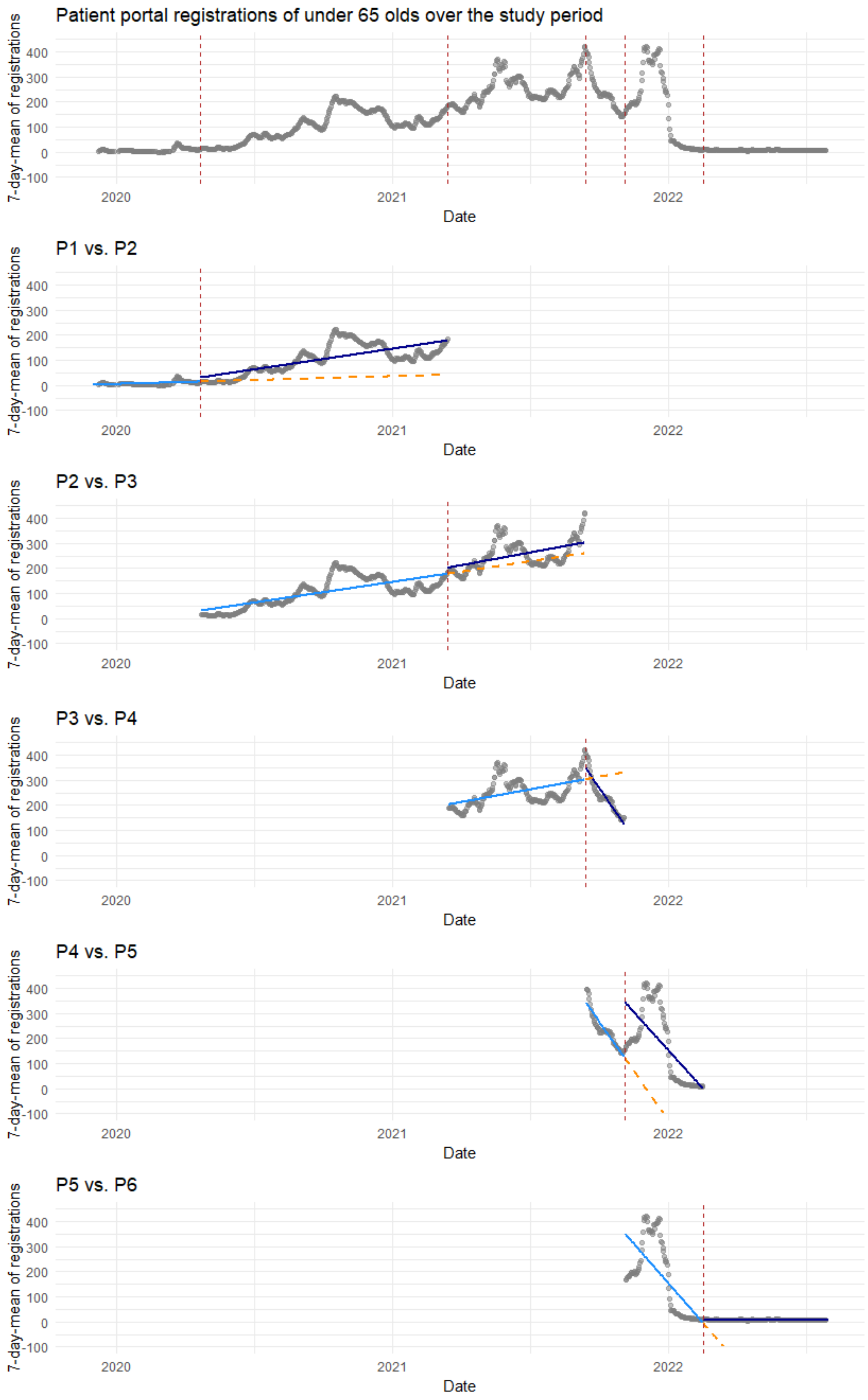

Figure S7. Interrupted time series analysis for each transition from one period to the next for under 65 olds.

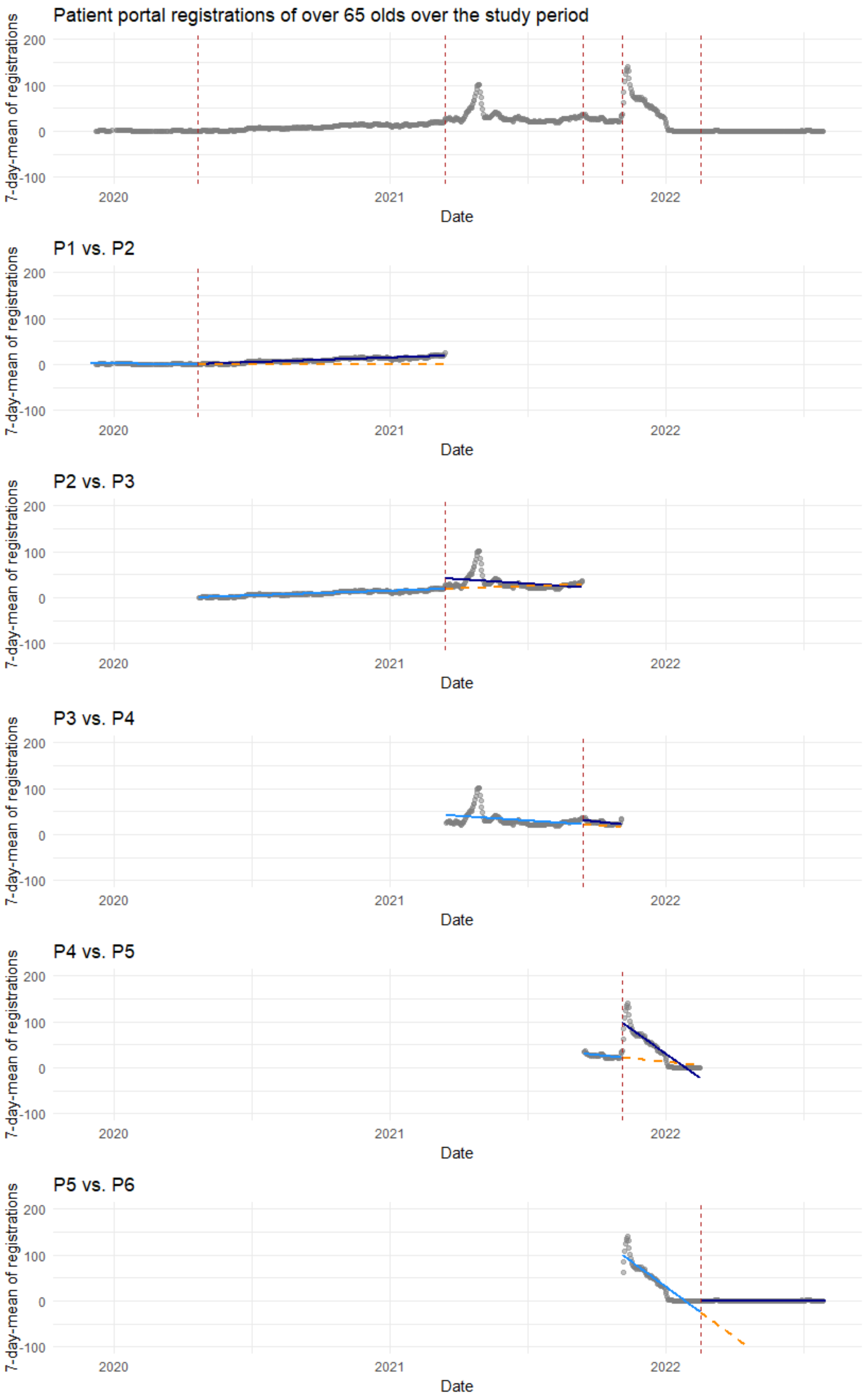

Figure S8. Interrupted time series analysis for each transition from one period to the next for over 65 olds.

Patient portal registrations grouped by gender

Table S7. Interrupted time series analysis for each transition from one period to the next grouped by gender.

		Female			Male		
		**Coefficient**	**95% CI**	***P***	**Coefficient**	**95% CI**	***P***
P1 vs. P2	Intercept	0.70	[-6.53, 7.92]	0.85	2.56	[-4.64, 9.76]	0.48
	Slope (pre-intervention trend)	0.06	[-0.02, 0.15]	0.14	0.02	[-0.07, 0.10]	0.69
	Level change (immediate effect)	5.53	[-2.02, 13.08]	0.15	10.18	[2.65, 17.70]	0.01
	Slope change (sustained effect)	0.22	[0.14, 0.31]	< .001	0.20	[0.12, 0.29]	< .001
P2 vs. P3	Intercept	-25.30	[-33.84, -16.76]	< .001	-16.13	[-24.96, -7.30]	< .001
	Slope (pre-intervention trend)	0.28	[0.26, 0.31]	< .001	0.22	[0.19, 0.25]	< .001
	Level change (immediate effect)	22.46	[14.07, 30.85]	< .001	24.41	[15.74, 33.09]	< .001
	Slope change (sustained effect)	-0.11	[-0.18, -0.04]	0.002	0.05	[-0.02, 0.12]	0.160
P3 vs. P4	Intercept	49.26	[11.02, 87.50]	0.01	-12.66	[-53.40, 28.09]	0.54
	Slope (pre-intervention trend)	0.17	[0.11, 0.24]	< .001	0.27	[0.19, 0.34]	< .001
	Level change (immediate effect)	31.66	[16.49, 46.83]	< .001	28.34	[12.18, 44.51]	< .001
	Slope change (sustained effect)	-2.52	[-2.97, -2.08]	< .001	-2.52	[-3.00, -2.05]	< .001
P4 vs. P5	Intercept	1677.10	[1070.05, 2284.15]	< .001	1607.13	[1034.96, 2179.30]	< .001
	Slope (pre-intervention trend)	-2.28	[-3.18, -1.39]	< .001	-2.18	[-3.03, -1.34]	< .001
	Level change (immediate effect)	154.13	[122.20, 186.07]	< .001	144.94	[114.84, 175.04]	< .001
	Slope change (sustained effect)	-0.02	[-0.97, 0.92]	0.96	-0.02	[-0.91, 0.87]	0.97
P5 vs. P6	Intercept	1905.99	[1734.08, 2077.91]	< .001	1818.31	[1656.70, 1979.91]	< .001
	Slope (pre-intervention trend)	-2.38	[-2.61, -2.15]	< .001	-2.27	[-2.48, -2.06]	< .001
	Level change (immediate effect)	18.97	[1.76, 36.19]	0.03	17.28	[1.09, 33.46]	0.04
	Slope change (sustained effect)	2.38	[2.12, 2.63]	< .001	2.27	[2.03, 2.51]	< .001

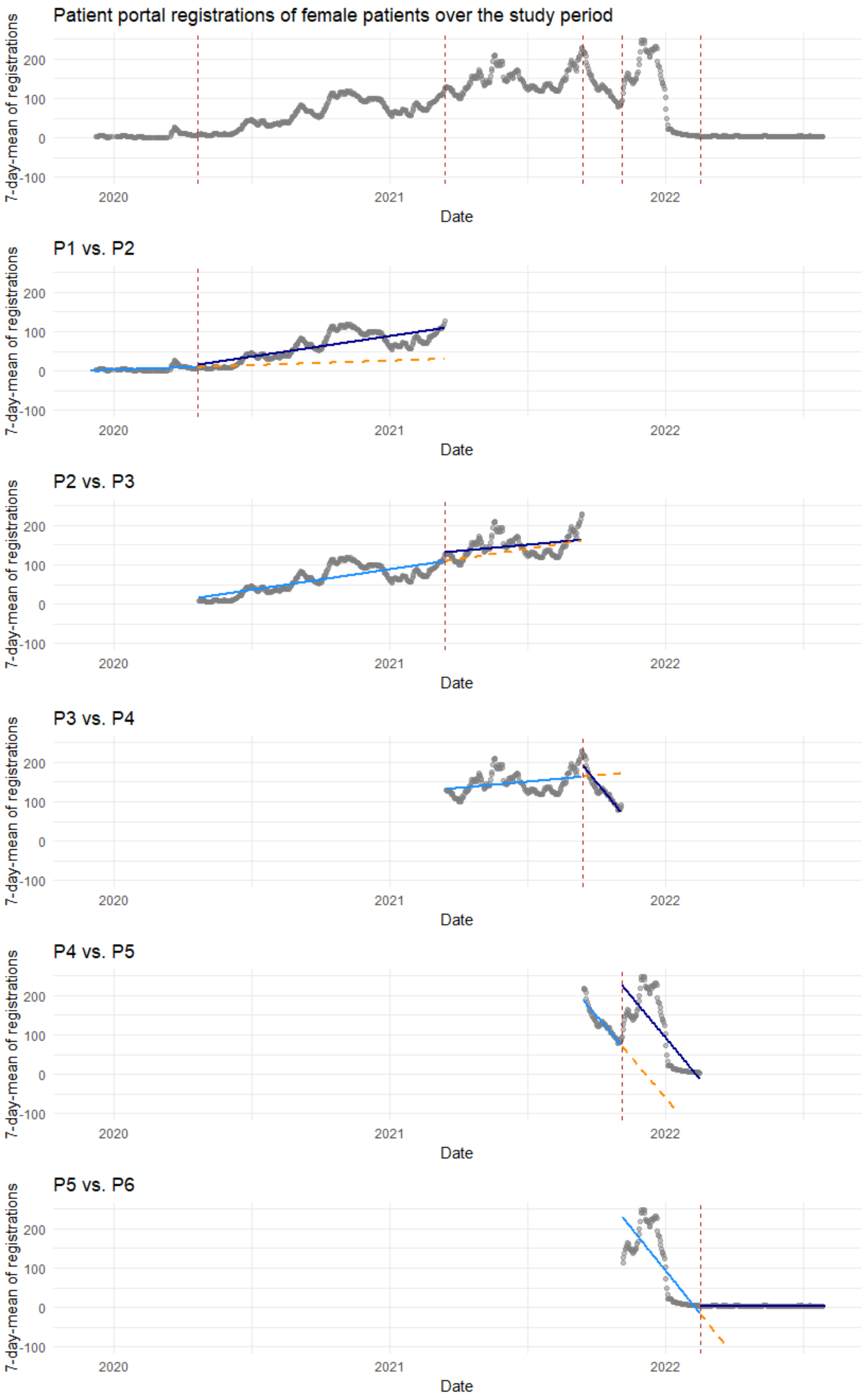

Figure S9. Interrupted time series analysis for each transition from one period to the next for female patients.

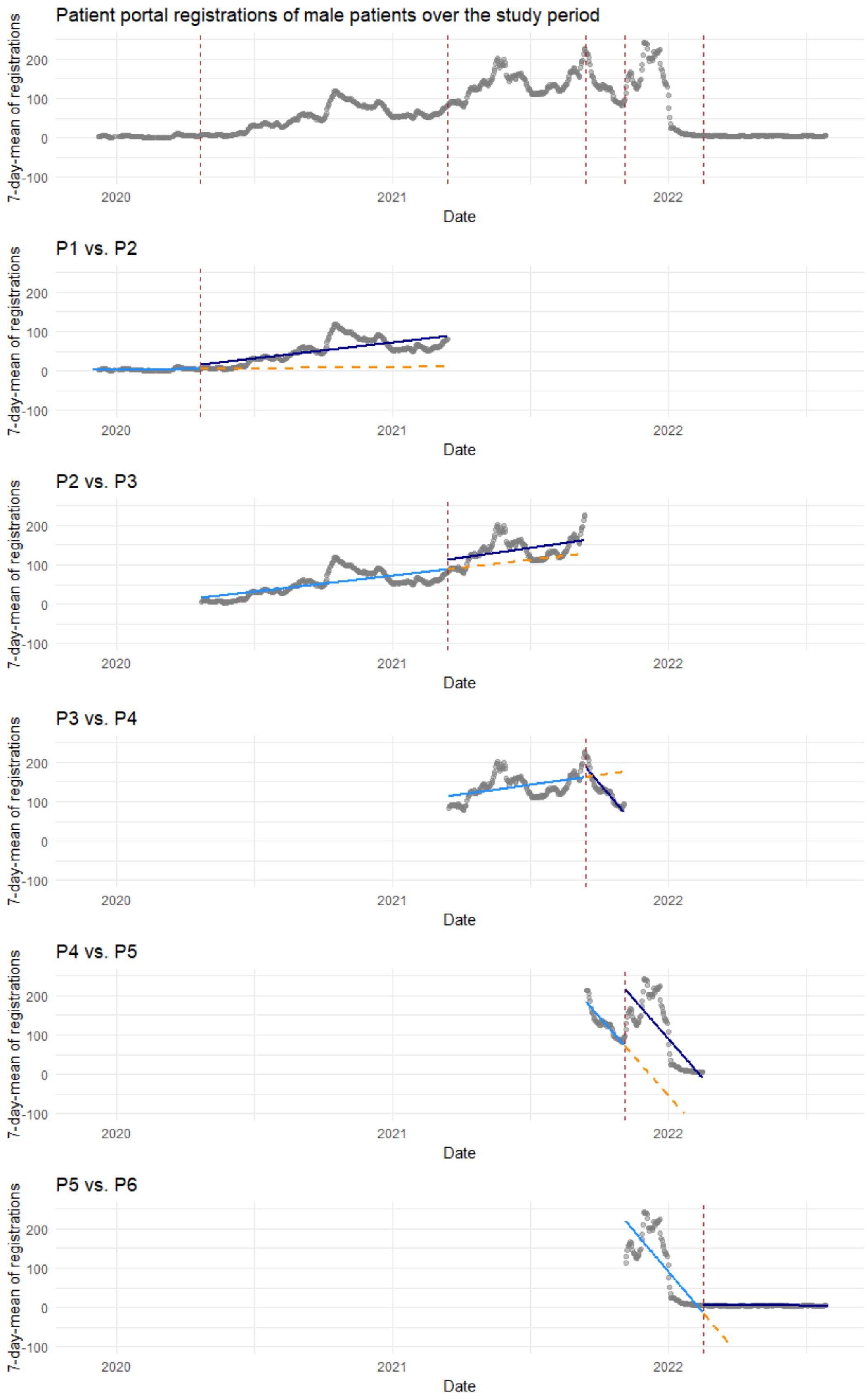

Figure S10. Interrupted time series analysis for each transition from one period to the next for male patients.
